# Penal Sanctioning of Zoophilia in Light of the Legal Status of Animals—A Comparative Analysis of Fifteen European Countries

**DOI:** 10.3390/ani10061024

**Published:** 2020-06-12

**Authors:** Szilvia Vetter, Anita Boros, László Ózsvári

**Affiliations:** 1 Department of Veterinary Forensics and Economics, University of Veterinary Medicine Budapest, 1078 Budapest, Hungary; vetter.szilvia@univet.hu (S.V.); ozsvari.laszlo@univet.hu (L.Ó.); 2 Globalization Competence Center, Széchenyi István University, 9026 Győr, Hungary; 3 Lajos Lőrinc Institute of Administrative Law, Faculty of Science of Public Governance and Administration, National University of Public Service, 1083 Budapest, Hungary

**Keywords:** animal welfare, zoophilia, legal status of animals, animal cruelty, penal sanctioning

## Abstract

**Simple Summary:**

The aim of the research is to examine the legal status of animals and the criminal law regulation of zoophilia (commonly referred to as “bestiality”) in 15 European countries. These two factors were chosen because they show how societies relate to animals through legislation, that is, how much animals are protected because of their inherent value, and not just because of the interests of humans. In the case of their legal status, the study examined the shift from viewing animals as a simple legal object to giving them special legal status. This research also examined criminal law definitions related to zoophilia. The results of compiling and comparing country rankings for zoophilia and legal status show that countries that place greater emphasis on regulating zoophilia are also more likely to have clearer rules in place regarding the legal status of animals. Switzerland is a positive example of both factors, while Italy faces many challenges in establishing specific legislation.

**Abstract:**

The criminal legislation regarding zoophilia and the legal status of animals were examined in 15 European countries. With regard to zoophilia, answers to the following questions were sought: are sexual acts performed with animals and the possession and distribution of animal pornography criminally punishable? Several aspects of the legal status of animals were examined including: (1) is the protection of individual animals included in the constitution, (2) do animals have a special status beyond mere objects, (3) can we find specific legislative provisions that explicitly state that animals are not simply things, and (4) does the legal system also take the “dignity” of animals into consideration. The assessment of zoophilia and the legal status of animals resulted in two country rankings, which the authors compared with each other. The correlation was not significant (*p =* 0.3147). At the same time, countries with differentiated criminal legislation for zoophilia were also 3.62 times more likely to rank higher in terms of the legal status of animals. The Swiss regulations are exceptional in both respects, while at the other end of the list, Italy does not have specific legislative provisions for either aspect.

## 1. Introduction

Zoophilia or bestiality, that is, sexual attraction to animals is as old as mankind [1]; in antiquity it was already a known phenomenon [2,3]. Its acceptance has varied throughout the course of history, for example, a god in the shape of an animal seducing a woman is a recurrent motif of ancient mythology and depictions of zoophilia can be found in countless works of art [4]. Following their initial acceptance, sexual relations with animals were severely punished. The Code of Hammurabi (dated to the 18th century BC) prescribed the death sentence for those involved [3]. Later, in the Middle Ages zoophiles were burnt at the stake together with the “guilty” animals [5]. Today, most societies condemn zoophilia, but those attracted to animals form secret subcultures. In their opinion, zoophilia is simply a sexual desire that is different from the norm. They report love and respect for the animals and invoke the proper conditions in which animals are kept [6].

Zoophilia is understudied, and usable statistics about the frequency of bestiality cases are limited or not available at all [7]. It is classified among paraphilias, a group of sexual disorders, where stimuli, which are considered to be unusual, are required for arousing sexual desire [8]. The World Health Organisation classifies zoophilia under “Other disorders of sexual preference”, a category which belongs to the “Disorders of adult personality and behaviour” within “Mental and behavioural disorders”. This classification is under revision [9]. The fifth DSM (Diagnostic and Statistical Manual of Mental Disorders) published by the American Psychiatric Association defines zoophilia as follows: “recurring and intensive sexual desire, which is … targeted at animals” [10]. Zoophilia and bestiality are used as synonyms; however, some researchers define zoophilia at the level of intention, with bestiality referring to the act taking place [11]. Attempts have also been made to popularise the far more value-neutral term, “zoosexuality” [12].

“Negative mattering’’ means all actions or events that harm animals [13], but some acts of zoophilia do not belong in this category. Legal regimes exist where criminal law does not punish acts motivated by zoophilia that cannot be considered cruelty to animals, for example, because they do not involve physical pain or other harm [14]. Naturally, this does not mean that these countries do not sanction zoophilia in other ways (for example, as a misdemeanour), only that the perpetrator is not punished through the use of criminal law. 

Those fighting for the strict sanctioning of zoophilia often invoke the lack of the “sufferer’s” (quasi “victim’s”) consent by the animal. In criminology, these expressions are reserved exclusively for humans; therefore, this reasoning is ideological rather than legal. According to Roman law, “volenti non fit injuria”, that is, acts committed with the consent of the victim are not unlawful. At present, the consent of the victim presents an obstacle to criminality, and if it does not harm the interests of society, this can be accepted as a line of defence [15]. The consent of the victim can also be regarded as a question of self-determination [16]. If we accept, theoretically, the “victim” status of the animal, unlawfulness can be established by the fact that an animal never gives, or can give, its consent to zoophilic acts. With people, sexual acts without consent are considered rape. In the case of animal pornography, economic interests may also be involved in addition to sexual preference [17].

In societies organised by the state, ensuring the rights of humans has been a central issue for millennia. Constitutional rules represent the foundation, theoretical basis, and also the limitations of national legislation, and in certain cases, international rules. While a large number of constitutions regulate certain biodiversity or health issues, with animals often appearing as resources and symbols, in the past few decades, provisions that recognise the intrinsic value of animals have also been introduced to some constitutions [18]. The main argument of those campaigning for the inclusion of animal welfare in the constitution is that it would prevent other rights set out in the constitution (e.g., the right to artistic freedom, to freedom of religious observance) to undeservedly over-rule animal welfare issues. Including animal protection in the constitution, therefore, produces an “equality of arms” between animal welfare and other constitutional rights.

Animals do not have legal capacity; only humans can be the subject of fundamental rights and freedoms. Today, in Europe, fundamental human rights are simultaneously protected at the country level, by the European Union, and the European Convention [19]. Since the 1970s, efforts have been made to shift the legal status of animals to that of a sort of “pseudo-entity” [20]. A breakthrough in legal opinion is yet to come (apart from limited legal subjectivity for apes [21]), but the need for change in the status of animals based on recognition of their intrinsic value has been acknowledged.

In a legal sense, only people can have a right to dignity [19]. The question of the “dignity of animals” is the subject of constant debate. It may be understood to refer to animals deserving dignity on account of their existence alone [18]. “Dignity” has traditionally been tied to human-centred or, at least, person-centric values [22]. A group of researchers have questioned the raison d’être of animal dignity [23,24]. According to Zuolo, the extension of dignity to animals is not appropriate, but the recognition of the moral importance of animals and their defence should appeal to other normative concepts [25]. 

Other authors believe that the existence of “animal dignity” is beyond doubt [26,27,28]. Ortiz goes so far as to state that respect for animal dignity provides a defeasible reason not to engineer an animal, even if the modification would improve the animal’s welfare [29]. However, in the absence of the legal personality of animals, a reference to their dignity is essentially contextless, although it provides highly indicative guidance from the legislator and expresses a respectful attitude towards animals.

The aim of our study was to examine the level of differentiation in criminal law regarding zoophilia and its legal status according to constitutional and civil law in 15 European countries: Hungary, Austria, Czech Republic, Denmark, France, Germany, Italy, The Netherlands, Norway, Poland, Slovakia, Slovenia, Spain, Sweden, and Switzerland. A further aim was to determine if the criminal legislation regarding zoophilia is in line with the legal status of animals. The basis of comparison is the common root of the two legal phenomena: the recognition of the inherent value of animals derived from being living creatures, and the capture of these values by the means of law. Both factors show how a given society (through legislation) relates to the added value that distinguishes a sentient animal from other non-sentient things. The question is to what extent the common ideology behind the two legal institutions result in similarly evolving legislation.

## 2. Materials and Methods 

During our examination of zoophilia, we considered those elements of criminal law that distinguish zoophile acts from cruelty to animals. The question is how criminal law can formulate rules to cover acts that are not necessarily cruelty to animals in the strict meaning of this term in criminal law, but are nonetheless motivated by zoophilia. In our study we only examined the provisions of criminal law [30,31,32,33,34,35,36,37,38,39,40,41,42,43,44,45,46,47,48,49,50]. In some countries it is not the penal code itself but other legislation (such as legal acts related to animal welfare) that provides for criminal sanctions in relation to animal cruelty and even zoophilia. The study examines the legislature that envisions the possibility of a custodial sentence (as the most severe punishment today) for these offenses.

The four aspects (questions) examined were:

1. Are certain forms of sexual acts performed with animals punishable?
2. Are all sexual acts performed with animals punishable?
3. Is the distribution of animal pornography punishable?
4. Is the possession of animal pornography punishable?

The examination of the legal status of animals also covered four elements. The criterion relating to constitutional status examined whether the protection of animals is included in the constitution of the country in some form [51,52,53,54,55,56,57,58,59,60,61,62,63,64,65,66,67,68,69]. Animals do not have legal capacity in any of the countries examined. However, nuances regarding object status can be found, which infer that major ideological differences are at play in the background. First, we examined whether animals have any special status beyond that of mere object and whether we can find reference-level regulations that relate to this (typically in the civil codes [70,71,72,73,74,75,76,77,78]). Second, we sought specific legislative provisions that clearly and explicitly state that animals are not simply objects in the legal sense. By the rules of logic, countries that meet the second criterion also meet the first. The last criterion examines whether the legal regime also considers the “dignity” of animals, as evidenced by specific legislative provisions.

Binary answers (yes/no) were given in response to the questions: 1 represents a positive, and 0 represents a negative answer. The criteria were divided into two groups: the first group (“Zoophilia”) is based on the legislation on zoophilia, the second (“Legal status”) contains the four aspects related to legal status. In both groups, total scores were converted to rankings by ranking the countries with a higher score more highly. This produced two country rankings that assessed zoophilia and the legal status of animals. The two rankings were compared using the following methods:

1. A parameter was created and expressed as a percentage. For each country, the two rankings were multiplied (their place in the rankings according to zoophilia/legal status), then the multiplicative inverse of the result was multiplied by 100.
2. The countries examined were classified into two groups according to both legal status and zoophilia: high ranking (1st, 2nd, 3rd place) and low ranking (4th and 5th place). The resulting legal status and zoophilia groups were compared with Fisher’s exact test. The analysis was performed in Version 3.6.1 of the R software [79].

## 3. Results and Discussion

**Figure 1:**
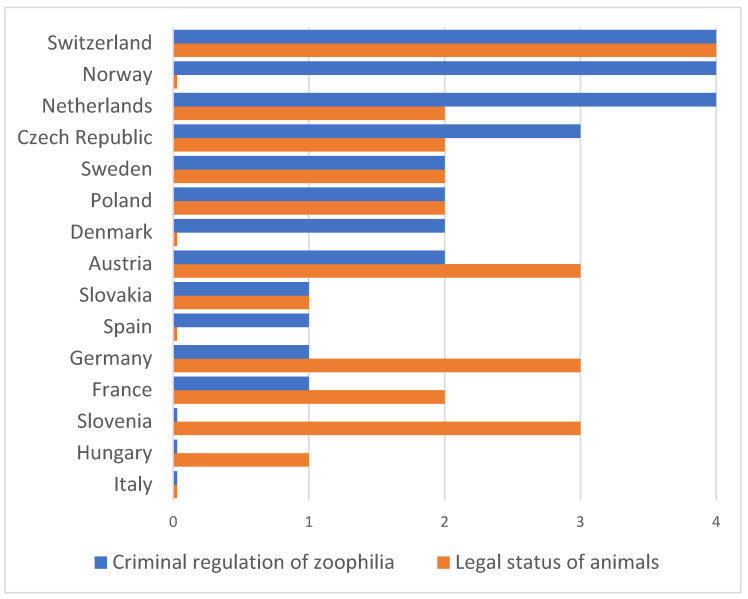
Ranking of 15 European countries according to the level of criminal regulation of zoophilia and the legal status of animals (based on the sum of values, from the most complete legal regulation (4) to the absence of regulation (0)).

The data collected from legal sources are summarized and converted to figures with binary coding in Table 1 for zoophilia and in Table 2 for legal status. The 15 countries were ranked according to the level of their legal status and criminal legislation on zoophilia. The total score for each country is presented in Figure 1.

### 3.1. Zoophilia

As shown, the criminal legislation of the Netherlands, Norway, and Switzerland is the most complete in terms of its assessment and sanctioning of zoophilia. At the other end of the country ranking are Hungary, Italy, and Slovenia as their criminal law does not include specific penal sanctions for cases of zoophilia. The remaining countries rank between these two extremes on the scale.

### 3.2. Legal Status of Animals

With respect to the legal status of animals, and its basis in constitutional law, we found that there was specific protection of animals in some form in five of the 15 countries (Austria, Germany, Slovenia, Switzerland, Sweden). The Austrian federal constitution lists the protection of animals in its legislation as a federal responsibility, although execution of this legislation is a state responsibility, unless otherwise provided for in federal legislation (Article 11. Paragraph 1. Point 8 [52]). In Germany, the relevant provision is contained Article 20a, according to which, in the context of constitutional orders and from a sense of responsibility for future generations, the State protects the natural foundation of life and animals [58]. The Slovenian constitution, in Article 72 on the right to a healthy environment, prescribes that the protection of animals from cruelty must be regulated in legislation [65]. The most detailed constitutional regulation of animal welfare is found in Switzerland. Pursuant to Article 80 the State has a legal obligation with regard to animal welfare. This obligation covers animal care, animal testing and procedures performed on live animals, the use of animals, the importation of animals and products of animal origin, trade in animals, the transport of animals, and the slaughter of animals. According to cardinal rule, it is the task of cantons to enforce these regulations [62]. Uniquely, the Swedish constitution provides protection against the depiction of violence against animals in the media. Article 11 provides a mandate to enact legislative provisions against video recordings or recordings made using other technical means that depict violent acts or threats against people or animals, and which are aimed at minors. In many cases, unlawful images of violence against animals are also a breach of the legislation [63]. Although this solution still falls short of protecting animals for their intrinsic value alone, and appears to be aimed more at protecting society, we consider it more forward-looking than specimen protection, or at the other extreme, completely omitting cruelty to animals from the constitution.

The first criterion concerning legal status in its narrowest sense (i.e., progressing from the mere “object” legal status of animals) was satisfied by 10 countries, and the “stricter” criterion (this being explicitly stated in the constitution) was met by eight countries. While Hungary [66] and Sweden [76] only received scores for the first criterion, the relevant regulations in Austria [67], the Czech Republic [68], France [70], Germany [72], the Netherlands [71], Poland [48], Switzerland [75] and Slovakia [77] clearly grant animals some sort of special object status. The third category contains countries where animals have mere object status in written law.

The Hungarian regulations are an example of compliance with the first criterion. Although it does not state that animals are not things, the Hungarian Civil Code provides as follows: “The rules on things shall apply to animals, taking the provisions of Acts establishing derogations reflecting their special nature into account” (Section 14, paragraph 3. [66]). Sweden does not have a civil code, and the terms “person” and “thing” are not defined within the law. Nevertheless, the spirit of the Swedish Animal Welfare Act, and the declaration that animals are subject to "respect" (Section 1, [76]), suggests that animals enjoy a special property status. However, this is not declared.

Eight of the examined countries meet the second criterion. According to the Austrian Civil Code, "Animals are not things; they are protected by special laws" (Paragraph 285, Point a [67]). As a result of the 2015 amendment to the French Civil Code, the following was declared, “animals are living beings endowed with sensitivity” (Article 515-14 [70]). The German Civil Code states that “Animals are not things. They are protected by special laws (Paragraph 90a [72])”. The same provision ("animals are not things") can be found in the Dutch Civil Code (Section 3.1.1, Article 3:2a [71]), and also in the Swiss Civil Code (Article 641a [75]). Under Article 494 of the Czech Civil Code “a living animal, as a sentient being, holds special value. Live animals are not things, and provisions concerning objects may only be applied to a live animal by analogy as long as they are not contrary to its nature [58]. The Polish Animal Protection Act states "the animal as a living creature, capable of suffering, is not a thing" (Chapter 1, Article 1.1 [48]). In Slovakia, an amendment that elevated animals from “object“ status and created the category “living beings” came into force in September 2018. The Civil Code of Slovakia now states that live animals enjoy special status, as they are able to perceive the world that surrounds them through their own senses. The provisions relating to chattels apply to live animals, unless this is contrary to the nature of the live animal as a living being (Section 119 (3) [77]).

Italy, for example, does not meet any criterion for the legal status of animals, even in the narrow sense. In its chapter on objects (Chapter II), the Italian Civil Code does not explicitly mention animals. The law divides objects into two categories: “immoveable property” and “chattel”. Article 812 defines immoveable property (land, trees, buildings, etc.) and stresses that all other objects are to be considered chattels, including (since they are not mentioned) animals [73].

None of the legislation examined outlines in detail and/or establish a new, independent legal category for animals, instead this is left to the interpretation of those who apply the law. Even if we accept that a new legal category has appeared that lies between objects and entities, it remains evident that it is closer to objects. For example, legislation prescribes the use of rules applicable to objects whereas there is no special rule applicable to animals.

The right to human dignity protects the essence of humankind, and could be considered the most important fundamental right, or the “parent” right of all other fundamental rights. Since current legal systems do not recognise animals as legal entities, their right to dignity is difficult to establish. Again, the exception is Switzerland, where legislators attempted to introduce the legal concept of animal dignity. In practice, the protection of animal dignity in the Swiss constitution, which is unique globally, means mostly that it is forbidden to humiliate animals, use them as tools, or alter their appearance [80]. An explicit provision to this effect cannot be found anywhere else.

In connection with legal status, that is, constitutional law, taking the two criteria relating to legal status in a narrow sense, and the dignity of animals, we can conclude that Switzerland has the most complete regulation, with Austria, Germany, and Slovenia lagging slightly behind. At the other end of the country ranking scale, we can place Denmark, Italy, Norway, and Spain, because with repect to legal status, the answer was “No” to both criteria for these countries.

### 3.3. Comparison of the Country Rankings According to Zoophilia and Legal Status

**Figure 2:**
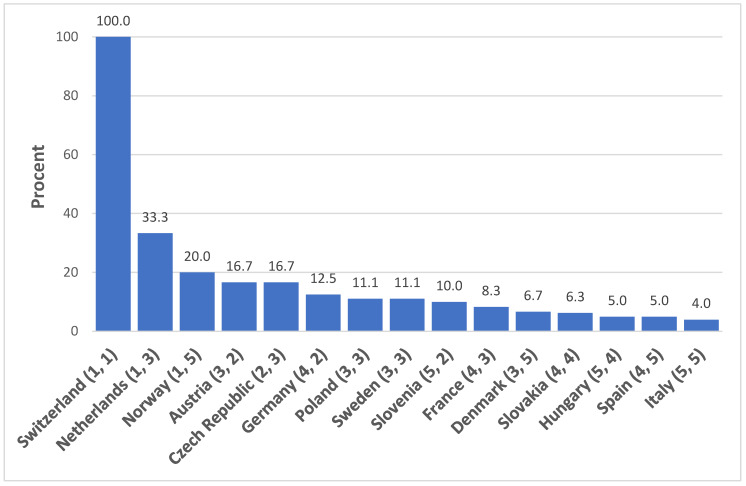
Evaluation of 15 European countries on the basis of the legal status of animals and the criminal aspects of zoophilia (next to the countries, the two rankings are shown in parentheses).

We then compared the ranking according to the regulation of the legal status of animals with that according to the form in which zoophilia appears in criminal law. To each country we assigned the two values (rankings) that illustrate the differences between the level of differentiation for zoophilia and the legal status in that country (Figure 2). The difference between the two rankings is noticeably high: four ranking places for Norway, three for Slovenia, and two for the Netherlands, Germany, and Denmark. There was no differences in the case of Switzerland, which leads both lists, third-placed Poland and Sweden, fourth-placed Slovakia, and Italy at the bottom. For all other countries the difference is one place.

In addition, we classified each country with a value expressed as a percentage. The higher this value, the more differentiated and “better” the legal regime of the given country for both legal status and zoophilia. (For example, for the Netherlands we obtained 33.3% as follows: we multiplied the ranking determined according to the level of regulation of the legal status of animals (3) with the ranking determined according to the level of regulation of zoophilia (1), then multiplied the multiplicative inverse of the value (1/3) with 100.) While Switzerland obtained the maximum score (100%), Hungary (5%), Spain (5%), and Italy (4%) are significantly behind in the differentiation of the regulation.

**Figure 3:**
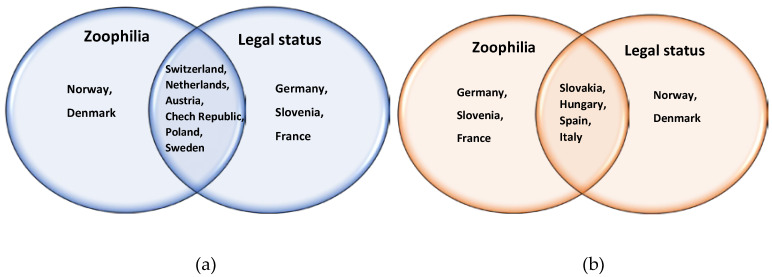
Rankings of the examined countries according to the legal status of the animals and the regulation of zoophilia. (**a**) High-ranking (1–3) countries; and (**b**) low-ranking (4–5) countries).

By statistical analysis we compared the legal status and zoophilia groups with Fisher’s exact test. We present the distribution according to legal status and zoophilia groups in Figure 3. The correlation between legal status and zoophilia was not significant (*p* = 0.3147). This result is due to the fact that, although in one-third of the countries examined (Switzerland, Sweden, Poland, Slovakia, and Italy) the regulations of the two aspects produced the same ranking, in another one-third of the countries (Norway, Netherlands, Czech Republic Denmark, and Spain) the more progressive response to zoophilia is not accompanied by an equally clear definition of legal status, while in the last one-third of the countries (Austria, Germany, France, Slovenia, and Hungary), the situation is the exact opposite, that is, the definition of the legal status of animals is more progressive than the response to zoophilia. At the same time, countries that regulate zoophilia more strictly were 3.62 times more likely to be ranked higher according to legal status; therefore it can be assumed that countries that place greater emphasis on regulating zoophilia are also more likely to have more detailed and clearer rules in place regarding the legal status of animals.

The recent improvement and qualitative change in the legal status of animals is not an isolated phenomenon; it is connected with the “animal revolution”, which can be observed in the philological and social sciences, and in popular culture and thought. Countries with geographical, historical, or political similarities follow a similar approach to animal welfare. Interest in animals and the relationship between animals and people is growing, and the emphasis has shifted to the subjective side of animals. Their physiological and individual experiences have also become morally, socially, politically and legally significant [18].

Public opinion on cases that are motivated by zoophilia, but do not qualify as cruelty to animals in the legal sense, is highly revealing of society’s approach to the “sui generis” values of animals [81]. The issue of animal “dignity” arises in cases where the sexual activity performed with animals does not cause physical harm to the animal and cannot, therefore, be classified as cruelty to animals. Some countries, even though they do not mention the dignity of animals in their constitution or laws (with the exception of Switzerland), take it into account when sanctioning cases motivated by zoophilia that do not result in impaired health. The dignity of animals is one of the main arguments of those campaigning for severe sanctions for zoophilia; therefore, in an ideological sense, the two aspects are closely related.

## 4. Conclusions

Overall, the current criminal approach to zoophilia and the animals’ legal status is highly varied in Europe, with different rules in each country. The Swiss legislation is exceptional in both respects, while at the other end of the list, Italy does not have specific legislative provisions on either issue. Among the 15 countries examined, we suggest there is a positive relationship between the legislation of the two factors, criminal sanctioning of zoophilia and modification of the legal status of animals. This leads to the conclusion that there may be a third variable that affects both to some extent. This third variable is the common ideological ground, that is the legal recognition of the intrinsic value of animals.

While the legislation regarding certain sexual acts is now clear (consensual homosexuality is no longer an act that is prohibited in Europe, but sexual acts performed with minors or based on violence remain forbidden), the legal view of zoophilia remains varied. The handling of the situation is made more difficult by the fact that, in the absence of reliable studies or statistics, the prevalence of zoophilia can only be estimated. For the most part, zoophilia remains a social taboo even where it is not forbidden, as the person involved will generally not advertise their preference. Sometimes, even animal welfare organisations are reluctant to address this topic.

Moreover, it is often observed that the steps required to change animal welfare regulations follow the same chronological line. Changes in public opinion are likely to lead to legal changes that affect animals, sooner or later. A clear trend has emerged that points towards differentiation and tightening of the law. The need for new rules typically reaches the world of politics at the behest of the society and civilians, and these are transmitted through the media (or social media) before such rules are eventually enacted as legislation. Many cases have emerged that have shocked society and accelerated legislation, and similar cases are likely to emerge in the future. The incidence of zoophilia is suspected to be considerable, but the cases that come to light cause a public outcry. In the long run, therefore, even countries currently without penal sanctions for zoophilia are likely to legislate more strongly against it in the future. Following the same line of reasoning, a further, more pronounced shift from the mere “object” legal status of animals and the introduction of specimen protection is also expected in those countries where this has not taken place yet. It is important to recognise that animal welfare is inseparably and symbiotically connected with the physical and mental health of humans.

## Figures and Tables

**Table 1:** Evaluation of 15 European countries according to the criminal regulation of zoophilia. ^1.^

Countries	Are Certain Forms of Sexual Acts Performed with Animals Punishable?	Are all Sexual Acts Performed with Animals Punishable?	Is the Distribution of Animal Pornography Punishable?	Is the possession of Animal Pornography Punishable?	Sum of Values
Austria	1	0	1	0	2
Czech Republic	1	1	1	0	3
Denmark	1	1	0	0	2
France	1	0	0	0	1
The Netherlands	1	1	1	1	4
Poland	1	0	1	0	2
Hungary	0	0	0	0	0
Germany	0	0	1	0	1
Norway	1	1	1	1	4
Italy	0	0	0	0	0
Spain	1	0	0	0	1
Switzerland	1	1	1	1	4
Sweden	1	1	0	0	2
Slovakia	0	0	1	0	1
Slovenia	0	0	0	0	0

^1^ Note: 1—yes, 0—no.

**Table 2:** Evaluation of 15 European countries according to the legal status of animals.^1.^

Countries	Is Animal Protection Included in the Constitution?	Special Status Compared to Simple “Thing” Status	Special Status Compared to Simple “Thing” Status-Specific Legislation	Specific Legislation of the “Dignity” of Animals	Sum of Values
Austria	1	1	1	0	3
Czech Republic	0	1	1	0	2
Denmark	0	0	0	0	0
France	0	1	1	0	2
The Netherlands	0	1	1	0	2
Poland	0	1	1	0	2
Hungary	0	1	0	0	1
Germany	1	1	1	0	3
Norway	0	0	0	0	0
Italy	0	0	0	0	0
Spain	0	0	0	0	0
Switzerland	1	1	1	1	4
Sweden	1	1	0	0	2
Slovakia	0	1	0	0	1
Slovenia	1	1	1	0	3

^1^ Note: 1—yes, 0—no.
